# A Deep Learning Framework for Predicting Patient Decannulation on Extracorporeal Membrane Oxygenation Devices: Development and Model Analysis Study

**DOI:** 10.2196/48497

**Published:** 2024-02-02

**Authors:** Joshua Fuller, Alexey Abramov, Dana Mullin, James Beck, Philippe Lemaitre, Elham Azizi

**Affiliations:** 1 Vagelos College of Physicians and Surgeons Columbia University New York City, NY United States; 2 Department of Surgery Columbia University Irving Medical Center New York, NY United States; 3 Clinical Perfusion New York Presbyterian Hospital New York, NY United States; 4 Department of Biomedical Engineering Columbia University New York City, NY United States; 5 Irving Institute for Cancer Dynamics Columbia University New York, NY United States; 6 Department of Computer Science Columbia University New York, NY United States; 7 Data Science Institute Columbia University New York, NY United States

**Keywords:** extracorporeal membrane oxygenation, ECMO, venovenous, VV, machine learning, supervised learning, dynamic data, time series, clinical decision support, artificial intelligence, AI, clinical AI, health informatics

## Abstract

**Background:**

Venovenous extracorporeal membrane oxygenation (VV-ECMO) is a therapy for patients with refractory respiratory failure. The decision to decannulate someone from extracorporeal membrane oxygenation (ECMO) often involves weaning trials and clinical intuition. To date, there are limited prognostication metrics to guide clinical decision–making to determine which patients will be successfully weaned and decannulated.

**Objective:**

This study aims to assist clinicians with the decision to decannulate a patient from ECMO, using Continuous Evaluation of VV-ECMO Outcomes (CEVVO), a deep learning–based model for predicting success of decannulation in patients supported on VV-ECMO. The running metric may be applied daily to categorize patients into high-risk and low-risk groups. Using these data, providers may consider initiating a weaning trial based on their expertise and CEVVO.

**Methods:**

Data were collected from 118 patients supported with VV-ECMO at the Columbia University Irving Medical Center. Using a long short-term memory–based network, CEVVO is the first model capable of integrating discrete clinical information with continuous data collected from an ECMO device. A total of 12 sets of 5-fold cross validations were conducted to assess the performance, which was measured using the area under the receiver operating characteristic curve (AUROC) and average precision (AP). To translate the predicted values into a clinically useful metric, the model results were calibrated and stratified into risk groups, ranging from 0 (high risk) to 3 (low risk). To further investigate the performance edge of CEVVO, 2 synthetic data sets were generated using Gaussian process regression. The first data set preserved the long-term dependency of the patient data set, whereas the second did not.

**Results:**

CEVVO demonstrated consistently superior classification performance compared with contemporary models (*P*<.001 and *P*=.04 compared with the next highest AUROC and AP). Although the model’s patient-by-patient predictive power may be too low to be integrated into a clinical setting (AUROC 95% CI 0.6822-0.7055; AP 95% CI 0.8515-0.8682), the patient risk classification system displayed greater potential. When measured at 72 hours, the high-risk group had a successful decannulation rate of 58% (7/12), whereas the low-risk group had a successful decannulation rate of 92% (11/12; *P*=.04). When measured at 96 hours, the high- and low-risk groups had a successful decannulation rate of 54% (6/11) and 100% (9/9), respectively (*P*=.01). We hypothesized that the improved performance of CEVVO was owing to its ability to efficiently capture transient temporal patterns. Indeed, CEVVO exhibited improved performance on synthetic data with inherent temporal dependencies (*P*<.001) compared with logistic regression and a dense neural network.

**Conclusions:**

The ability to interpret and integrate large data sets is paramount for creating accurate models capable of assisting clinicians in risk stratifying patients supported on VV-ECMO. Our framework may guide future incorporation of CEVVO into more comprehensive intensive care monitoring systems.

## Introduction

### Background

Extracorporeal life support (ECLS) is a suite of resource-intensive therapies indicated in patients with refractory respiratory failure or cardiogenic shock [1]. This intervention involves cannulation of central or peripheral arteries and veins to provide forward flow through a circuit with a mechanical pump and gas exchange device, also called a membrane oxygenator. Air is connected to the membrane oxygenator to deliver oxygen and remove carbon dioxide from the circulating blood. Established indications for venovenous extracorporeal membrane oxygenation (VV-ECMO) exist in the literature, and the use of this technology was expanded during the COVID-19 pandemic [2]. The VV-ECMO configuration is specifically used for patients experiencing severe lung injury. This setup is designed to provide oxygenation and decarboxylation support without offering the additional hemodynamic assistance found in the venoarterial configuration. VV-ECMO is considered a last resort therapy for patients with end-stage respiratory failure [3], with an overall survival rate of 60% [4].

Decannulating a patient from VV-ECMO is a clinical challenge that requires considerable training and expertise from provider teams in the intensive care unit. Clinicians assess trends in the patient’s vital signs, physical examination, response to various therapies, laboratory biochemistries, and radiographic studies. When the decision is made to proceed, decannulation is usually accomplished through a weaning trial during which VV-ECMO is gradually reduced. To date, there are limited prognostication scores that successfully predict when patients are ready to undergo a weaning trial. In this study, we present an artificial intelligence model capable of running in real time that incorporates discrete and continuous variables that clinicians may use in their assessment of patients for decannulation from VV-ECMO support.

### Related Work

Multiple predictive scores have been developed to help clinicians prognosticate before cannulation. The 6 most common prognostication scores for adult respiratory failure supported on extracorporeal membrane oxygenation (ECMO) are ECMOnet, Predicting Death for Severe Ards on VV-ECMO, Respiratory ECMO Survival Prediction, Roch, Venovenous ecmo mortality score, and Prediction of Survival on ECMO Therapy score [5] (Table S1 in Multimedia Appendix 1 [6-11]). Although these 6 scores are commonly used, they have 2 main drawbacks. First, all input information is recorded before cannulation to ECLS because the primary intent of the models is to be used to determine which candidates were most likely to benefit from the intervention. Second, all scores use logistic regression to predict outcomes or identify significant variables. Logistic regression requires high-quality data from static variables, which limits the types of data that can be inputted. Thus, sequential or time-series data such as laboratory values and vital signs must be limited to a single time point or summarized. Furthermore, these statistical models are limited in terms of capturing nonlinear effects and interactions between variables.

To date, no studies have mitigated both issues to improve the prediction of successful decannulation in patients supported on VV-ECMO. However, some researchers have attempted to use deep learning to predict specific clinical events.

Abbasi et al [12] used clinical and ECLS data to compare 2 approaches, deep learning and traditional statistical methodology, to develop a model to predict hemorrhage and thrombosis events. The deep learning model outperformed linear regression in both hemorrhage and thrombosis data sets, suggesting that more complex models may achieve better predictive power. Other authors have applied deep learning and modified logistic regression to predict survival on venoarterial-ECMO (VA-ECMO) only. Ayers et al [13] used 48 hours of laboratory values after VA-ECMO cannulation to predict survival to discharge using a deep neural network.

Similarly, Loyaga et al [14] used clinical, echocardiographic, laboratory, and hemodynamic characteristics to predict 30-day mortality in patients on VA-ECMO using the elastic-net method. None of these studies used data obtained from the ECMO devices, and instead used laboratory values, clinical scores, and disease severity to train their models. These approaches leave a large amount of valuable information unused. In the hospital, clinicians adjust the parameters of ECMO support in real time according to the patient’s condition and pathophysiology. Modern devices capture the interplay between the patient and ECMO by continuously collecting perfusion data [15]. Analysis of information-dense perfusion data may be leveraged to improve the prediction accuracy of clinically meaningful outcomes in ECLS care.

Incorporating more granular data requires a new model that is capable of integrating categorical and time-series data. The prevalence of recurrent neural networks (RNNs) in health care data science has increased recently. The ability of RNNs to efficiently understand time dependencies makes this approach beneficial in certain types of medical data, such as ventilator settings [16], vital signs [17], medication administration [18], imaging studies [19], and radiology reports [20]. One type of RNN, long short-term memory (LSTM), is specifically designed for long time series, such as our data set with weeks-long hospital courses. LSTM can encode these time series into a compressed latent space, which can be concatenated with static variables, such as age, gender, and other clinical characteristics.

### Novelty

The innovation of our study is two-fold: (1) data source and (2) algorithm design. Perfusion data were collected from the ECMO devices and recorded at highly granular intervals. Our analysis sheds new light on the effectiveness of ECMO. Second, unlike prior work using laboratory values, clinical scores, and other static data, the patient information used in our study was both dynamic and static. Using a 2-headed neural network, our predictive algorithm efficiently incorporates static information, such as sex and clinical scores, along with dynamic data. LSTM networks encode the perfusion time-series data into a latent space, which is then concatenated with an encoding of the static variables. This new latent space was used to classify patients.

We present the Continuous Evaluation of VV-ECMO Outcomes (CEVVO) predictive model for determining successful decannulation from VV-ECMO using both pre- and postcannulation data. When using both, the model can continuously update its prediction, providing a running measure for patient potential recovery. Such a measure may help clinicians and patient families make more informed decisions about care. Using synthetic data sets, we demonstrate that understanding time dependence is the essential ingredient to accurate predictions. Our framework also guides the categorization of patients into high-risk and low-risk groups, alerting care providers about which patients may be better candidates for weaning trials and decannulation.

## Methods

### Problem Formulation

Health care data of this type can be presented in two components: (1) clinical information that remains unchanged over the ECMO course, such as age and sex, which are considered static features, and (2) variables that change over time, such as laboratory values and perfusion data, which are considered temporal variables. This study follows the conventions presented in the study by Yoon et al [21]. We define *S* as a vector space of static features, and *X* as a vector space of temporal features. Let S ∈ *S* and X ∈ *X* be random vectors with specific values denoted by s and x. Each patient is a tuple of (s, x_1:T_), where T is the number of time steps. For clarity, patients in our training set were indexed by *n* ∈ 1,...,N. Therefore, the training data set is denoted as D = (s_n_,x_n,1:T_)^N^_n=1_. Each patient also had a categorical outcome *y* ∈ {0,1}, which forms vector Y across all patients, with 0 representing unsuccessful decannulation and 1 representing success. We define the probability distribution p(Y|S,X_1:T_), and our goal is to use training data *D* to learn a density p̂(Y|S, X_1:T_) that best approximates p(Y|S,X_1:T_). This is achieved through the optimization in equation 1:

*Min_p̂_ D_KL_* (p(Y|S,X_1:T_) || p̂(Y|S, X_1:T_)) **(1)**

The abovementioned Kullback-Leibler divergence can be calculated through the loss function in equation 2. This is identical to the cross-entropy because the entropy of the ground truth distribution is 0. The model can best approximate the true distribution by using backpropagation to minimize equation 2:

*L =* (−1/*N*) ∑*^N^_n_*_=1_ (*y_n_ log*(ŷ*_n_*) + (1*−y_n_*) *log*(1− ŷ*_n_*)) **(2)**

### Synthetic Data Set

We hypothesized that the high performance of the LSTM-based architecture is owing to its superior ability to capture long-term dependencies in the data set. To test this notion, 2 synthetic data sets of size N=234 and *t*=2054 were generated using a Gaussian process regression (GPR) model [22]. As GPR is nonparametric, it can generate synthetic data without making assumptions about the underlying relationships between variables and dynamics over time. By tuning parameters of the generative model, we can adjust the strength of long-term dependencies in the data. Using the GPR model, we sample data from a multivariable normal distribution, in which the covariance encodes dependencies between time points as shown in equation 3:

ƒ~
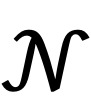
(μ, Σ) **(3)**

where denotes the expected values of the inputs and denotes the covariance. The covariance is encoded by a radial basis function (RBF) kernel, as shown in equation 4. The length scale parameter *L* of the RBF adjusts the local smoothing. Higher values for this parameter encode dependencies over a longer period, leading to smoother dynamics.

*k*(*x_i_,x_j_*) = *exp*(-(*d*(*x_i_,x_j_*)^2^/(2L)) **(4)**

where *d*(*x_i_,x_j_*) denotes the Euclidean distance. The long-term dependencies are captured by the probability of observing specific values conditioned on earlier time points. This assumption is reasonable in our application to VV-ECMO and not necessarily held in previous models such as logistic regression and some deep neural networks. The performance of previous models on GPR data is thus not affected by different choices of length scale, whereas the LSTM-based model should lose its advantage with increasing length scale.

Two groups of synthetic data were created: the first with *L*=1 and the second with *L*=100, and it was expected that CEVVO would be the only one to perform substantially better on *L*=1. The other models should have similar performance between *L*=1 and *L*=100. The length scale had to be larger than or equal to each time step; therefore, *L*=1 was close to the minimum allowable length scale.

### Model Design

**Figure 1:**
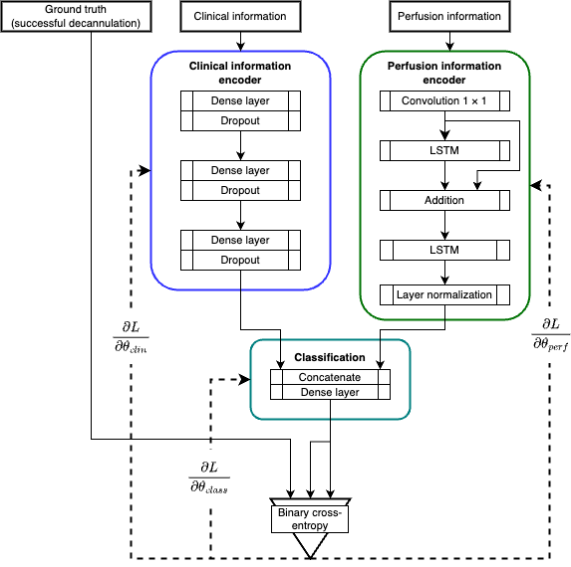
The overall architecture of the model. The double-headed approach allows the model to integrate static and dynamic data. Solid lines denote function application, and dashed lines denote loss computation. LSTM: long short-term memory.

A general overview of this framework is presented in Figure 1. It is composed of 2 independent heads: a static (clinical) data encoder and a temporal feature (perfusion) encoder. Theoretically, each distills the relevant information from the 2 data sets (clinical and perfusion) before concatenating them in the classification block.

The static information encoder is based on an autoencoding scheme along with an additional final dense layer. The first dense layer had 32 nodes, the second layer had 33, and the final layer had 25 nodes. These dimensions were chosen via Bayesian optimization hyperparameter tuning implemented through the *Keras* Python package by Chollet et al [23].

The perfusion information encoder was based on LSTM layers. These recurrent networks were found to work exceedingly well, as they were built on the assumption that earlier time points have marginal effects on later time points. A 1×1 convolutional layer was first used to expand the feature map before the LSTM to create a projection shortcut and act as a filter. The *tanh* activation function allowed the convolution layer to increase, decrease, or negate certain input values. Although an additional LSTM layer could do this processing, the convolution layer contained significantly fewer parameters. The filter size for the convolution and the LSTMs was 1024, which was also chosen via Bayesian optimization hyperparameter tuning.

The classification block concatenated the final outputs of the clinical information encoder and the perfusion encoder. By this point, the original clinical inputs were reduced from 32 to 25, and the original perfusion inputs were reduced from 16,432 to 1024. These 2 final layers were concatenated into a final layer of 1049. This led to a single output neurone with a *sigmoid* activation, which acted as the final prediction. This prediction was then compared with the ground truth, and the loss was calculated using the binary cross-entropy. The average of all the losses was calculated with equation 2. These losses were backpropagated through the network to make the probability distribution generated by the model resemble the reality.

### Defining Patient Risk Grouping

For the risk groups to have meaning, the calibration of the model must be assessed. A calibration plot for the training set was created and showed an S-shaped misalignment. The misalignment was corrected using Platt scaling.

Four clinical groups were defined with respect to the calibrated mean and SD of the model’s predictions on *D*. Let *M_D_* and *S_D_* be the mean and SD of the sigmoid output values of training data *D*. The grouping was determined according to equation 5:


| 0, if *x* <= *M_D_* -*S_D_*

| 1, if *M_D_* - *S_D_* < *x* <=*M_D_*

f_group_(x) = | 2, if *M_D_* < *x* <= *M_D_* + *S_D_*
**(5)**

*|* 3, if *M_D_* + *S_D_* < x

### Ethical Considerations

This study was conducted in accordance with the institutional review board of the Columbia University (#AAAT0563).

### Data

A retrospective chart review was performed, and continuous perfusion data and clinical information were collected from 118 patients cannulated to VV-ECMO at a high-volume ECMO center’s intensive care unit between January 1, 2020, and December 31, 2021. Patients reconfigured to venoaterial-venous or venoarterial were excluded.

**Figure 2:**
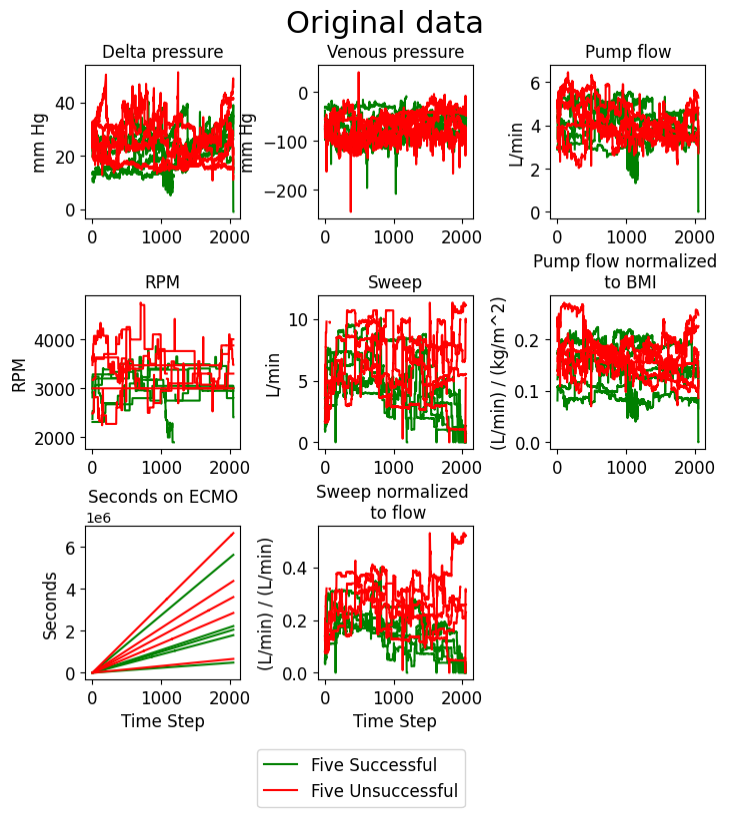
Extracorporeal membrane oxygenation (ECMO) perfusion data for 5 example patients with successful (green) or unsuccessful (red) decannulation. RPM: revolutions per minute.

Patient data were collected from Spectrum Medical software (Quantum Informatics), which records data from each patient’s ECMO machine. Six relevant perfusion variables were selected with expert insight and were collected at 120-second intervals. These were the pressure change across the membrane lung, the venous drainage pressure, the blood flow across the ECMO circuit, the pump head rotation speed (needed to generate the blood flow), the sweep gas flow (rate of oxygenated gas flowing through the membrane lung), and length of time the patient was supported on ECMO. Two additional perfusion variables were created to account for differences between patients: the flow across the pump divided by the patient’s BMI and the sweep gas flow divided by the flow across the pump. In addition to these 8 perfusion variables, 12 clinical variables were selected: decannulation result, age, sex, cause of respiratory distress, BMI, cardiac arrest before ECMO, shock (ie, hemodynamic instability) before ECMO, reinfusion and drainage cannulation location, reinfusion and drainage cannula size, and the type of ventilation provided (Table S2 in Multimedia Appendix 1). The 12 clinical variables included the outcome label, which was not included in the input data. Further clinical information that was not included in the model can be found in Table S3 in Multimedia Appendix 1. An example of 5 successful and 5 unsuccessful patients is shown in Figure 2. The chaotic nature of the perfusion variables helps to justify more advanced machine learning methods.

To enable incorporation of all time points in each VV-ECMO run, the first preprocessing step involved truncation, which refers to clipping the perfusion data set at different percentages of the total run. For each patient, in addition to the 100% of the ECMO run (ie, the full run), the first 90%, the first 80%,..., the first 10% of the run were appended as additional runs. Thus, the full data set involved 1180 sequences of data points, 10 for each patient. Each data point consisted of a 3D perfusion time series (patient deidentified ID code, time step, and variable) and 2D clinical data (patient deidentified ID code and variable).

Owing to varying ECMO run lengths, each time-series sequence was standardized to 2054 time steps. This length was the largest size possible, given the GPU constraints. Standardization was performed by averaging dense time steps and forward-filling empty steps. The remaining empty time steps were set to 0. Truncations were treated as full runs, that is, the final values for the 10% and the 100% truncation occurred at the same time step—2053. Each truncation is, in effect, stretched over the 2054 time steps. This ensures that the model is not given hints about which truncation it is seeing.

The performance of the model was evaluated through cross-validation. In each iteration, the list of patient IDs was randomized and split into 5 groups of 23 patients, each with 18 successful and 5 unsuccessful patients. Three random patients were excluded to have 5 groups of the same size. Three groups of patients (69/115, 60% of the total) were chosen as the training data set, one group (23/115, 20%) was chosen as the validation set, and one group (23/115, 20%) was chosen as the test set. This process was repeated 5 times until each group had been included in the test set once. The patient list was then randomized again to begin the next cross-fold validation. This ensured that the training set, validation set, test set, and unused patients differed each time. In total, there were 12 iterations of this 5-fold cross-validation.

Each set of training data consisted of 69 patients, and the validation and test sets had 23 patients. Including all truncations, the training set had 690 data points, and the validation and test sets had 230 data points each.

The data sets were then scaled using MinMaxScaler from the *sklearn* Python package by Pedregosa et al [24]. The scaler was trained on the training data and then used to transform all 3 sets.

### Synthetic Data Set

**Figure 3:**
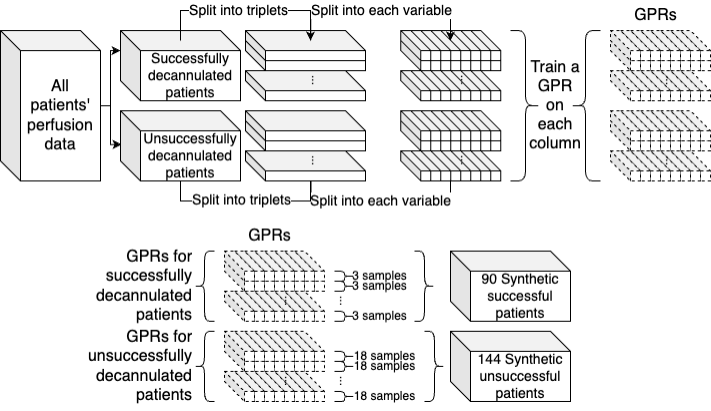
Diagram of the process of generating synthetic patient data. Solid boxes indicate patient data (including synthetic), and dotted boxes indicate a Gaussian process regression (GPR) model fit to real patient data. The sampling of the GPRs step was repeated 2 times, 1 for each kernel length scale.

The GPRs were generated using the Gaussian process regressor Python package *sklearn* Pedregosa et al [24]. Two different data sets were generated with different values for the length scale of the RBF kernel (1, 100). A GPR model was first fit to unnormalized perfusion data from all patients. To generate more realistic synthetic data, patients were divided into successful and unsuccessful decannulation groups and sorted according to the ECMO run time. They were then grouped into triplets based on these criteria, which resulted in 30 *successful* triplets and 8 *unsuccessful* triplets. To create the training data for the GPRs, each patient’s age, gender, and BMI were extracted and normalized using the StandardScaler from the *sklearn* Python package by Pedregosa et al [24]. These, in addition to a time-step value, were treated as independent variables. The dependent variable was the unnormalized perfusion data from the triplet. Each GPR was trained only on a single perfusion variable, so each triplet had 8 GPRs, 1 for each perfusion variable. For each of the 30 *successful* triplets, each GPR model was sampled 3 times for a total of 90 synthetic *successful* patients. For the 8 *unsuccessful* triplets, each GPR was sampled 18 times for a total of 144 synthetic *unsuccessful* patients. A diagram of this process is shown in Figure 3.

The triplets were then split into training, validation, and test sets in the same manner as the original patient data. For each iteration of the 5 cross-folds validation, the triplets were randomized and split into groups of 7, each with 6 *successful* triplets and 1 *unsuccessful* triplet. A random set of 3 (38%) *unsuccessful* triplets, out of 8, were not included in each interaction of the 5 cross-folds. This was done to guarantee that each group had the same number of triplets. Inside the groups, each of the 6 *successful* triplets yielded 3 synthetic patients, whereas the 1 *unsuccessful* triplet yielded 18 synthetic patients. This balanced out each group, with a total of 36 synthetic patients per group. Three groups were assigned to the training set, one group was the validation set, and the last group was the test set. Similar to the real data, the test set was rotated until each synthetic patient was tested.

### Model Assessment

After each model was trained, predictions were calculated for the test sets. Each prediction varied between 0 and 1 owing to the *sigmoid* activation in the final neurone. To assess performance, area under the receiver operating characteristic curve (AUROC) and the average precision (AP) were calculated using the *sklearn* package. AP approximated the area under the precision-recall curve. The predictions and ground truths were sampled 5000 times with replacement to create the AUROC and AP CIs. The bootstrapping pseudocode for estimating the AUROC CIs can be found in algorithm S1 in Multimedia Appendix 1. This bootstrapping code was then repeated for different subsets of the data. The AUROC and AP CIs were calculated for each day after cannulation between 0 and 24 (eg, the AUROC and AP for all data points ending on day 10, including truncations). These values were plotted along with their CIs. For the synthetic data, the bootstrapping method was only used on the entirety of each data set.

A successful model is expected to provide accurate and reliable insight into whether a patient will be decannulated.

## Results

### Model Performance on Real Data

The model’s overall performance on the real data achieved an average AUROC of 0.6937 (95% CI 0.6822-0.7055). The mean AP was 0.8599 (95% CI 0.8515-0.8682).

**Figure 4:**
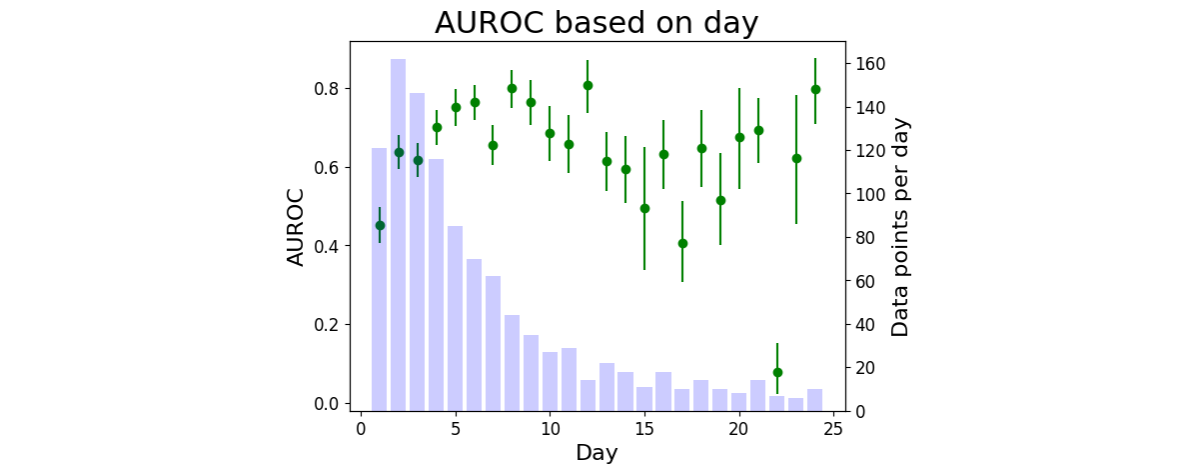
The area under the receiver operating characteristic curve (AUROC; in green) computed from all samples within a 1-day time frame, for example, AUROC for samples collected between days 0 and 1 are shown on day 1. Purple bars indicate the number of data points occurring on that day (right y-axis).

**Figure 5:**
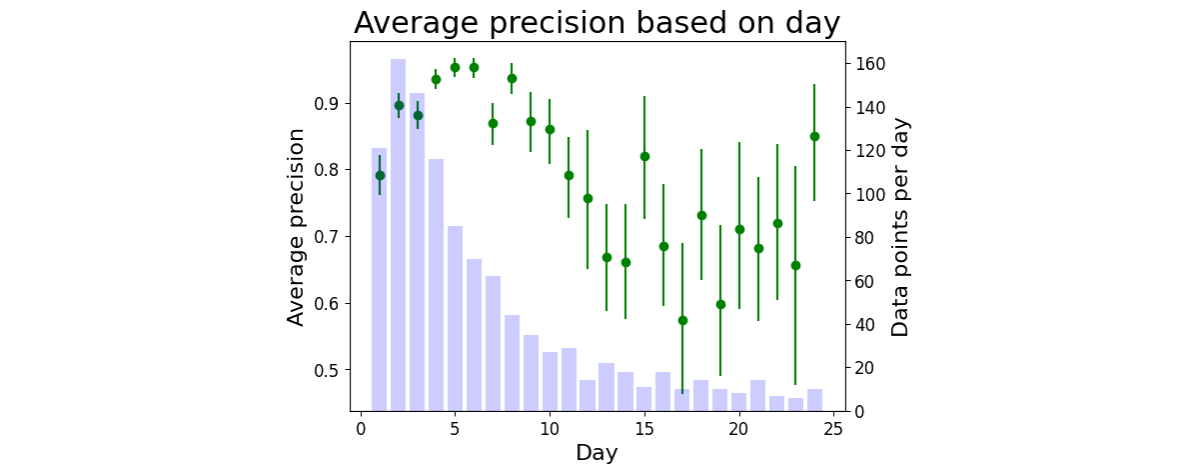
The average precision (AP; in green) computed from all samples within a 1-day time frame, for example, AP for samples collected between days 0 and 1 are shown on day 1. Purple bars indicate the number of data points occurring on that day (right y-axis).

A clinically relevant breakdown is AUROC and AP by day, as shown in Figures 4 and 5. Therefore, we observed that tight CIs begin to expand after day 11 as the number of data points decreased. By limiting the time frame to only include patient data points sampled between days 3 and 11, the AUROC 95% CI was 0.7048-0.7428, and the AP 95% CI was 0.9074-0.9261.

### Model Comparison on Real Data

As detailed in Table S2 in Multimedia Appendix 1, ECMOnet, Predicting Death for Severe Ards on VV-ECMO, Respiratory ECMO Survival Prediction, Roch, Venovenous ecmo mortality score, and Prediction of Survival on ECMO Therapy score rely on either logistic regression or recursive partitioning analysis to determine the patient grouping or scoring classification. To provide a fair comparison with the proposed model, the AUROC and AP calculations were repeated with a logistic regression model and a decision tree. Both models were trained on the same training data and assessed on the same test data as the proposed model. Moreover, the 95% CIs were determined with the bootstrapping algorithm presented in algorithm S1 in Multimedia Appendix 1. Furthermore, to provide a comparison with the study by Ayers et al [13], a dense neural network was included. Finally, a Naive Bayes model was included to demonstrate the necessity of including dependence between time points. Unlike the LSTM, Naive Bayes assumes conditional independence between features, making previous models incapable of understanding the time series as anything beyond a bag of values. Table 1 demonstrates that CEVVO is the most effective model for ECMO data, showing a significant improvement compared with other methods. Using a permutation test, CEVVO demonstrated a significantly higher AUROC (all *P* values <.001) and AP (all *P* values <.04) than all other methods.

**Table 1:** Comparison of Continuous Evaluation of Venovenous Extracorporeal Membrane Oxygenation Outcomes (CEVVO) with other models used previously.

Model name	Total AUROC^a^, 95% CI	Total AP^b^, 95% CI	*P* value compared with CEVVO (AUROC)	*P* value compared with CEVVO (AP)
CEVVO	0.6822-0.7055	0.8515-0.8682	—^c^	—
Logistic regression	0.6395-0.6626	0.8396-0.8566	<.001	.04
Naive Bayes	0.5876-0.6081	0.8111-0.8255	<.001	<.001
Dense network	0.5673-0.5908	0.8148-0.8322	<.001	<.001
Decision tree	0.5419-0.5596	0.5273-0.5467	<.001	<.001

^a^AUROC: area under the receiver operator characteristic.

^b^AP: average precision.

^c^Not applicable.

### Risk Classification System

**Figure 6:**
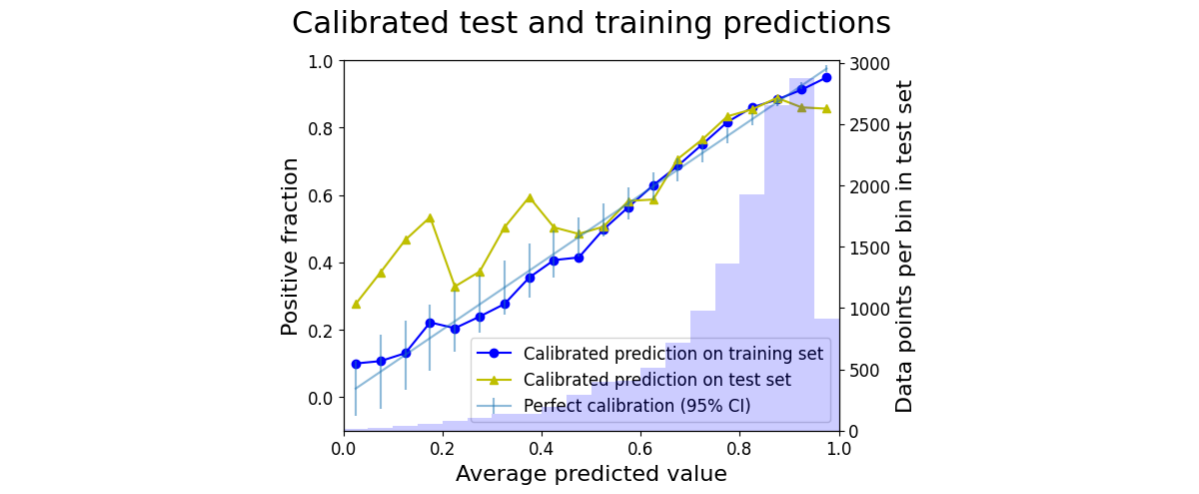
The calibration plot for both the training and test set prediction. Each set of predictions has been scaled. The green line shows the theoretical perfect calibration, and the purple bars show the number of data points in each bin.

The calibration plot of the training data is shown in Figure S1 in Multimedia Appendix 1. The classic S-shaped misalignment indicated that Platt scaling would improve the calibration. Both the calibrated training and test sets are shown in Figure 6.

**Figure 7:**
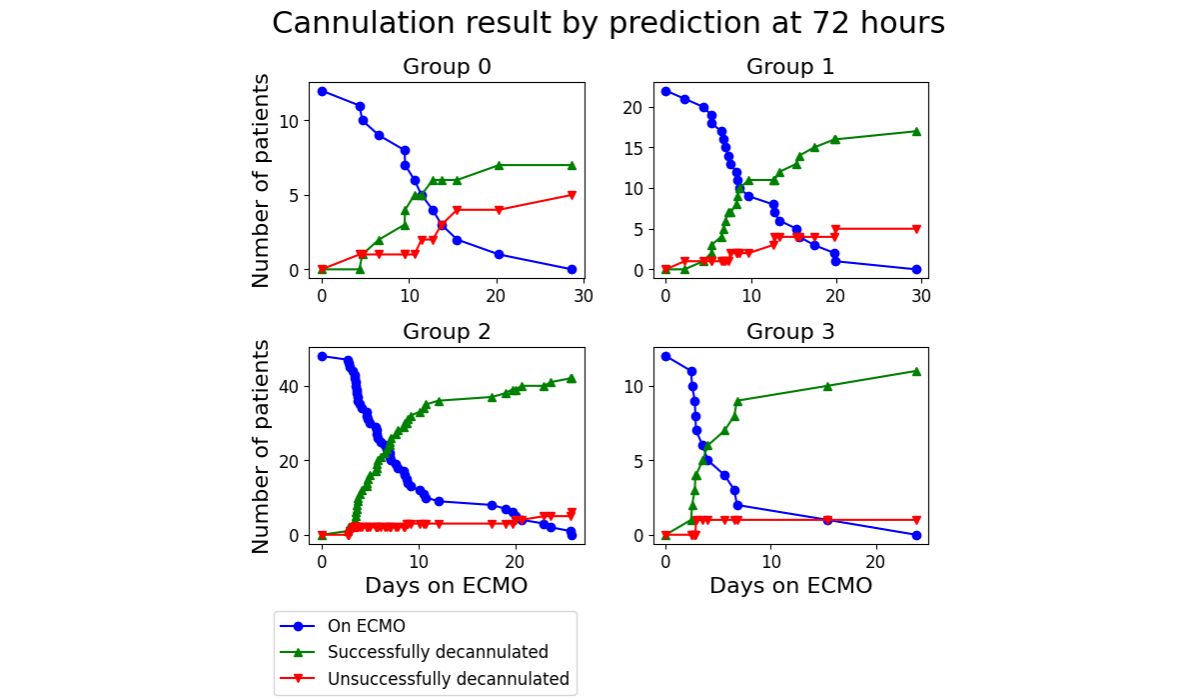
Patient result based on groupings at 72 hours. ECMO: extracorporeal membrane oxygenation.

**Figure 8:**
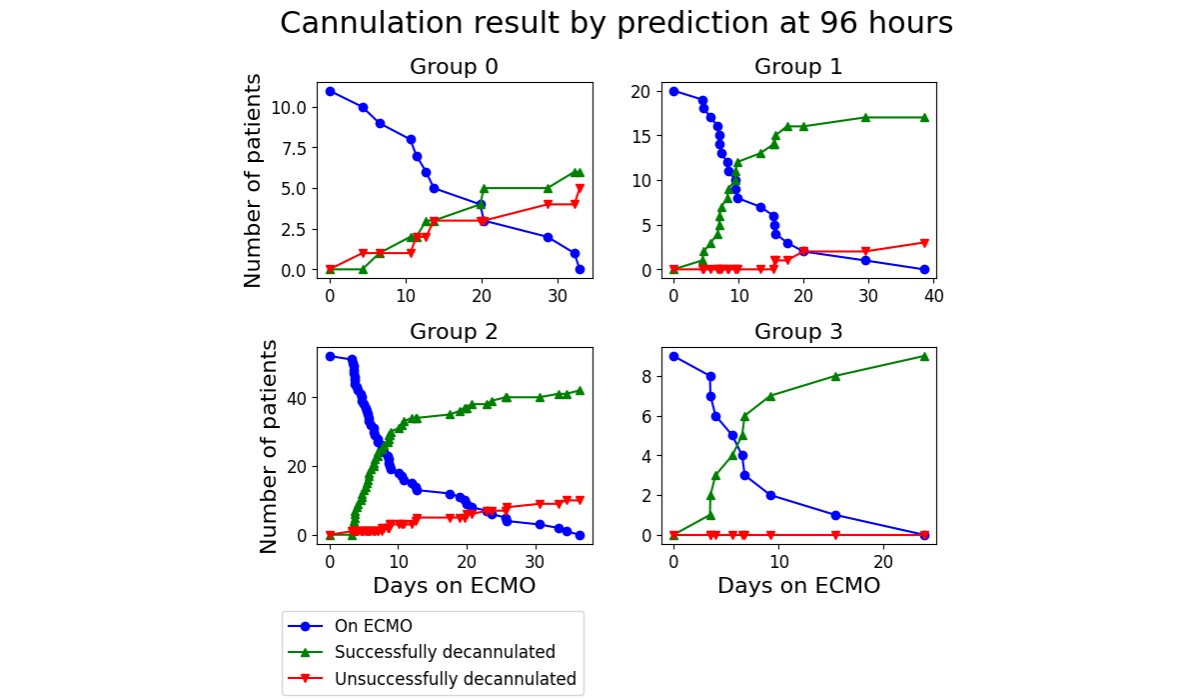
Patient result based on groupings at 96 hours. ECMO: extracorporeal membrane oxygenation.

Using the predictions as an indication of favorable or unfavorable outcomes, patients can be stratified into groups based on their prediction value using equation 5. The clinically relevant measures of performance are shown in Figures 7 and 8. These charts were created by finding the nearest predicted value of each patient before either 72 or 96 hours, sorting them into groups according to equation 5, and then charting their decannulation result. Patients decannulated before 72 or 96 hours were excluded. In the 72-hour case, the groups had a successful decannulation rate of 58% (7/12) for group 0, 77% (17/22) for group 1, 88% (42/48) for group 2, and 92% (11/12) for group 3. In the 96-hour case, the groups had successful decannulation rates of 54% (6/11), 85% (17/20), 81% (42/50), and 100% (9/9), respectively.

A Boschloo exact test between groups 0 and 3 yielded *P* values of .04 for 72 hours and .01 for 96 hours.

### Necessity of Time Dependencies

**Figure 9:**
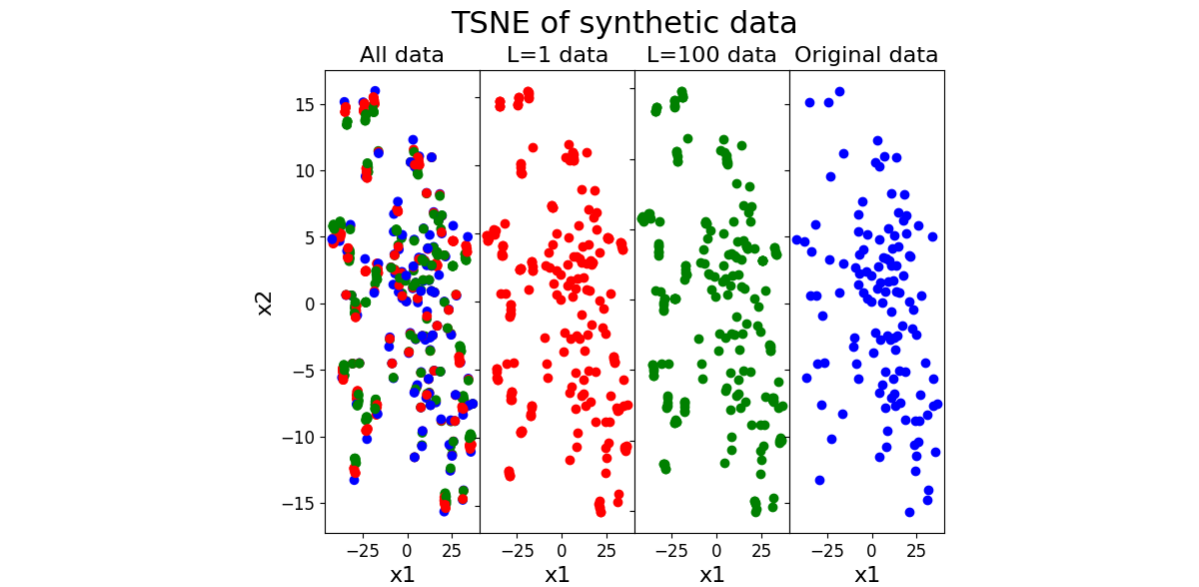
2D t-distributed Stochastic Neighbor Embedding (tSNE) of the 2 synthetic sequential data set and the original patient data. Each dot represents a synthetic patient; the red dots indicate data generated using a radial basis function (RBF) with L=1, the green dots indicate data generated using length L=100, and the blue dots indicate the original data. The significant overlap connotes similarity between the literal values.

**Figure 10:**
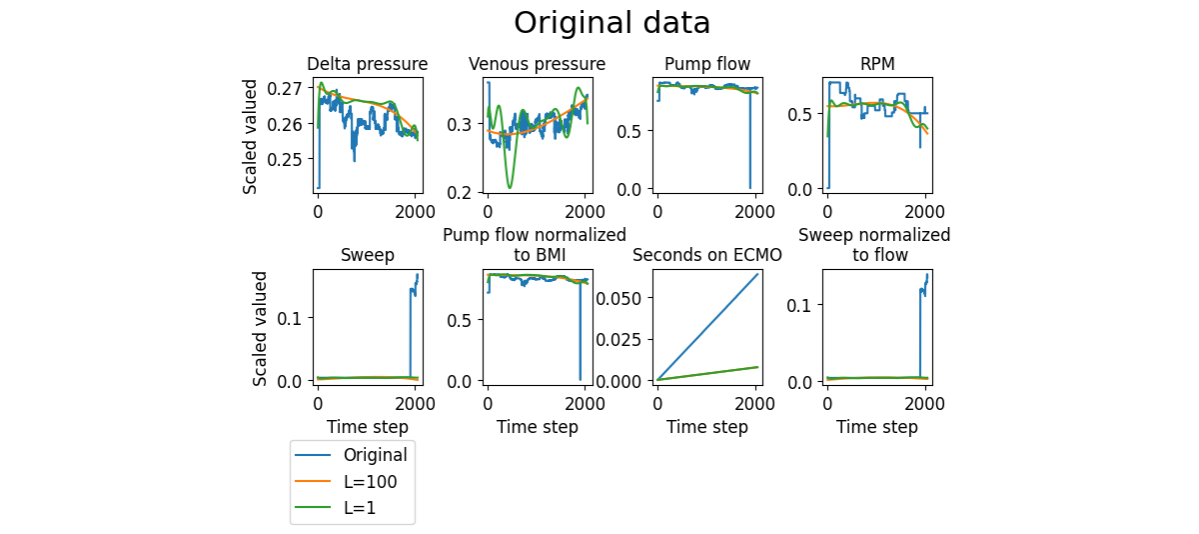
An example synthetic patient, shown in both the L=1 and L=100 data sets compared with a similar real patient (in blue). ECMO: extracorporeal membrane oxygenation; RPM: revolutions per minute.

To ensure that each of the synthetic data sets were comparable with each other and the original, a t-distributed Stochastic Neighbor Embedding [25] was used (Figure 9). A more concrete example is shown in Figure 10, where a single synthetic input was run through both the *L*=1 and *L*=100 GPRs and then compared with an original patient.

The procedure specified in the *Model Assessment* section was repeated for CEVVO, logistic regression, and dense network on the synthetic data set. The 95% CI for the AUROC is shown in Table 2. The expected result is observed where logistic regression and the dense network show no change in performance. CEVVO shows a significant drop in performance despite having similar, nonlinear properties to the dense network.

**Table 2:** Comparison of Continuous Evaluation of Venovenous Extracorporeal Membrane Oxygenation Outcomes (CEVVO) with top-performing models used previously on each synthetic data set.

Model name	Total AUROC^a^ for *L*=1 synthetic data set, 95% CI	Total AUROC for *L*=100 synthetic data set, 95% CI	*P* value between *L*=1 and *L*=100
CEVVO	0.8223-0.8583	0.7424-0.7849	<.001
Logistic regression	0.7813-0.8213	0.7814-0.8190	.46
Dense network	0.7080-0.7513	0.6924-0.7352	.17

^a^AUROC: area under the receiver operator characteristic.

## Discussion

### Principal Findings

VV-ECMO is an invasive and resource-intensive therapy used for patients with refractive respiratory failure. Decannulation from ECMO is generally performed through a weaning trial, in which the ECMO support, measured as flow through the circuit, is titrated down. Experienced clinician decision-making with careful consideration of patient hemodynamics, response to therapy, and pathophysiology informs the decision on when to perform the weaning trial. Our study investigates a novel approach to analyzing clinical information and perfusion hemodynamics in real time to assist clinicians with the decision of when to move forward with decannulation from VV-ECMO.

Although CEVVO was more accurate at predicting the success of decannulation than other models, the model should be considered as an additional data point to guide clinical management. Patients stratified to the high-risk group had a higher risk of therapy failure, with >50% of the patients in this group successfully decannulated in both the 72 and 96 hour cases. As expected, the calibration plot also showed that patients in the low-risk group were decannulated successfully more often. Using these data, clinicians may reference the model and elect to start weaning trials on patients stratified to the low-risk cohort sooner.

### Comparison With Prior Work

To the best of our knowledge, CEVVO is the first to use ECMO perfusion data and a deep learning architecture to provide clinical decision support for the decannulation decision for VV-ECMO. By using a model that can successfully combine dynamic and static data, significantly improved performance on binary classification can be achieved when compared with other models. Using perfusion data and clinical information, CEVVO was trained to classify patients by decannulation outcome (successful or unsuccessful). The performance was evaluated using 3 criteria: AUROC, AP, and the clinical usefulness of predictions. Relative to other models noted in the literature, such as logistic regression and decision trees, the LSTM-based model showed significant improvement on the ECMO machine data set.

### Performance

The AUROC and AP scores for the full data set had 95% CIs of 0.6822-0.7055 and 0.8515-0.8682, demonstrating a fair ability to predict exact outcomes. This was marginally improved to 95% CIs of 0.7048-0.7428 and 0.9074-0.9261 by limiting the data set to only consider data points collected 3 to 11 days after cannulation. However, these numbers only represented the average performance.

### Synthetic Data

The use of GPR-created data sets further cemented the notion that the novelty of the architecture, understanding time dependence, is truly what is responsible for the performance edge over other models. The assumption of temporal dependence is inherent in the data as it is medically motivated. There is an expectation that the specific value of the perfusion data shares much mutual information with the outcome. The L=1, L=100, and original data sets are very similar in their t-distributed Stochastic Neighbor Embedding projection, differing only slightly in the specific values. However, as shown in Figure 10, within the L=100 group, the local structure was obliterated, leading to a loss of information about how later time points affect the outcome probability. Logistic regression explicitly assumes that each time point is independent, and thus, it has highly similar AUROC distributions (*P*=.46) compared with the dense network (*P*=.17) and CEVVO (*P*<.001). The nonlinear nature of dense neural networks is able to approximate time dependence but is less efficient than the LSTM-based architecture.

### Risk Classification

These initial measures of performance were then used to contextualize the clinical predictions: stratifying people into groups, based on associated risk, to predict recovery. The numerical value of each patient’s prediction was divided into groups, and patients were followed to their decannulation result. For the grouping to be useful, there should be some difference in the success percentage that increases from the high-risk group to the low-risk group. This result was observed in this study. When measured at 72 hours, 58% (7/12) of the patients in the high-risk group had a successful decannulation, whereas 92% (11/12) of the patients in the low-risk group were successfully decannulated (*P*=.04). When measured at 96 hours, the successful decannulation percentage was similar: 54% (6/11) of the patients in the high-risk group and 100% (9/9) patients in the low-risk group were successfully decannulated (*P*=.01).

### Limitations

Cohort studies using retrospective data collection are subject to inherent bias. We mitigated bias in this study by including all consecutive patients supported on VV-ECMO at our center.

Incomplete data recording from the ECMO devices may have contributed to this model. In the future, this could be mitigated by increasing the sample size and improving data capture methodology.

Clinically, patients with different indications for ECMO support vary in their hospital course, and the number of different disease etiologies may have been too few for the model to learn. Larger cohorts may help mitigate the issues related to an underpowered data set. Furthermore, model performance declined beyond a period of approximately 11 days, which may be attributed to a challenging hospital course with heterogeneous factors and an increased risk for complications. The effectiveness of ECMO as a long-term therapy remains unclear, and our data support this conclusion.

### Future Direction

In the future, more information about each patient’s hospital course, such as administration of vasopressors, ventilator settings, imaging studies, and other interventions may be used to develop an improved model. Indeed, more data and reducing unaccounted variables may improve model performance over longer periods. Extending this study to include patients on other forms of ECLS, such as VA-ECMO and cardiogenic shock, may be helpful in guiding clinical management. We suggest that larger and more comprehensive repositories of health care data may improve the management of patients considered most critically ill.

## Appendix

Multimedia Appendix 1 Demographic data for the patient population, along with the bootstrapping algorithm.

## Abbreviations

AUROC: area under the receiver operating characteristic curve
AP: average precision
CEVVO: Continuous Evaluation of Venoarterial Extracorporeal Membrane Oxygenation Outcomes
ECLS: extracorporeal life support
ECMO: extracorporeal membrane oxygenation
GPR: Gaussian process regression
LSTM: long short-term memory
RBF: radial basis function
RNN: recurrent neural network
VA-ECMO: venoarterial extracorporeal membrane oxygenation
VV-ECMO: venovenous extracorporeal membrane oxygenation

## Data Availability

The main clinical data set is unavailable for the public because it contains protected health information. However, 2 synthetic data sets generated based on the original data are available at the Aizilab GitHub data set, along with the corresponding Python code.
