# Supplementary material for: A Deep Learning Framework for Predicting Patient Decannulation on Extracorporeal Membrane Oxygenation Devices: Development and Model Analysis Study
Source: JMIR Biomed Eng. 2024 Feb 2;9:e48497. doi: 10.2196/48497 (PMC11041448; doi:10.2196/48497)
Supplement: Multimedia Appendix 1 [file biomedeng_v9i1e48497_app1.docx]

*Appendix:*

Table S1: Previously reported ECMO Prognostication Scores

| Name | Target | Model/Method |
| --- | --- | --- |
| ECMONet | Overall Survival | Generalized Estimating Equation^6^ |
| Predicting dEath for Severe aRds on Vv-ECMO (PRESERVE) | Mortality six-months post ICU discharge | Logistic regression^7^ |
| Respiratory Extracorporeal membrane oxygenation Survival Prediction (RESP) | Hospital Mortality | Logistic Regression^8^ |
| Roch | Hospital Mortality | Recursive partitioning analysis^9^ |
| Veno-Venous (VV) ecmo mortality score | Hospital Mortality | Logistic Regression^10^ |
| PREdiction of Survival on Ecmo Therapy score (PRESET) | Hospital Mortality | Logistic Regression^11^ |

Table S2: Clinical Information by Decannulation Result

| Characteristic |  | Successful Decannulation (n=90) | Unsuccessful Decannulation (n=28) |
| --- | --- | --- | --- |
| Age (year) – mean (SD) |  | 42.44 (13.33) | 46.50 (11.78) |
| Male – no (%) |  | 58 (64.4) | 15 (53.6) |
| **Cause of ARDS – no (%)** |  |  |  |
|  | COVID | 24 (26.7) | 14 (50) |
|  | Bacterial Pneumonia | 19 (21.1) | 4 (14.3) |
|  | Flu | 15 (16.7) | 1 (3.6) |
|  | Aspiration | 12 (13.3) | 3 (10.7) |
|  | Non-Flu Viral Pneumonia | 6 (6.7) | 4 (14.3) |
|  | Drug Toxicity | 5 (5.6) | 0 (0) |
|  | Non-Pulm Sepsis | 3 (3.3) | 2 (7.1) |
|  | Fungal Pneumonia | 2 (2.2) | 0 (0) |
|  | Acute Rejection | 1 (1.1) | 0 (0) |
|  | Fat Embolism | 1 (1.1) | 0 (0) |
|  | Granulomatosis with  Polyangiitis | 1 (1.1) | 0 (0) |
|  | Transfusion-related acute  lung injury | 1 (1.1) | 0 (0) |
| BMI – mean (SD) |  | 32.89 (9.01) | 30.29 (9.47) |
| Cardiac Arrest Pre-  ECMO – no (%) |  | 11 (12.2) | 1 (3.6) |
| Shock Pre-ECMO – no  (%) |  | 69 (76.7) | 19 (67.9) |
| **Reinfusion Cannula Size – no (%)** |  |  |  |
|  | 20 | 60 (66.7) | 20 (71.4) |
|  | 22 | 14 (15.6) | 4 (14.3) |
|  | 18 | 6 (6.7) | 1 (3.6) |
|  | 21 | 5 (5.6) | 1 (3.6) |
|  | 19 | 2 (2.2) | 0 (0) |
|  | 15 | 1 (1.1) | 2 (7.1) |
|  | 27 | 1 (1.1) | 0 (0) |
|  | 31 | 1 (1.1) | 0 (0) |
| **Drainage Cannula Size – no (%)** |  |  |  |
|  | 25 | 38 (42.2) | 11 (39.3) |
|  | 23 | 37 (41.1) | 12 (42.9) |
|  | 29 | 7 (7.8) | 3 (10.7) |
|  | 21 | 4 (4.4) | 1 (3.6) |
|  | 27 | 3 (3.3) | 1 (3.6) |
| **Reinfusion Cannula**  **Location – no (%)** |  |  |  |
|  | Left Femoral Artery | 0 (0) | 1 (3.6) |
|  | Left Internal Jugular Vein | 29 (32.2) | 11 (39.3) |
|  | Right Femoral Artery | 1 (1.1) | 1 (3.6) |
|  | Right Femoral Vein | 7 (7.8) | 1 (3.6) |
|  | Right Internal Jugular Vein | 53 (58.9) | 14 (50) |
| **Drainage Cannula**  **Location – no (%)** |  |  |  |
|  | Left Femoral Vein | 35 (38.9) | 12 (42.9) |
|  | Right Femoral Vein | 53 (58.9) | 16 (57.1) |
|  | Right Internal Jugular  Vein | 2 (2.2) | 0 (0) |
| **Ventilation – no (%)** |  |  |  |
|  | Pressure Assist Control | 14 (15.6) | 2 (7.1) |
|  | Volume Assist Control | 69 (76.7) | 24 (85.7) |
|  | Airway Pressure Release  Ventilation | 3 (3.3) | 1 (3.6) |
|  | Pressure Regulated Volume Control | 1 (1.1) | 0 (0) |
|  | None | 3 (3.3) | 1 (3.6) |

Table S3: Clinical information not included in model

| Characteristic | Successful Decannulation (n=90) | Unsuccessful Decannulation (n=28) |
| --- | --- | --- |
| Mortality – no (%) | 6 (6.7) | 28 (100) |
| Days on ECMO – mean (SD) | 15.57 (18.24) | 23.57 (18.03) |
| Days in the Hospital – mean (SD) | 49.01 (41.17) | 28.61 (21.11) |

| **Algorithm S1:** Bootstrapping algorithm |
| --- |
| **Input:** Ground Truth *G*: *t_0_, t_1,_* …, *t_n_;* Predictions *P*: *p_0_,p_1_,…,p_n_* ; Repetitions *R*  /*AUROC is undefined if G doesn’t contain both classes  **If** *Ground Truth doesn’t contain at least one 1 and one 0* **then**  Return an Error  **End**  *A* ← ∅  **For** *x* = 0, 1, …, *R* **do**  Choose x as a seed for Randomizer  *N’* ← Randomly choose n integers between 0 and n with replacement  *G’* ← Values from *G* at indices *N’*  *P’* ← Values from *P* at indices *N’*  **While** *N’ doesn’t contain at least one 1 and one 0* **do**  Randomly rechoose *N’* and reassign *G’* and *P’*  **End**  *AUC* ← 0  **For** *all pairs (g_i_, g_j_) in G where g_i_ =* 1 *and* *g_j_ =* 0 **do**  **If** *p_i_ > p_j_* **then**  Add 1 to *AUC*  **End**  **End**  Divide *AUC* by the number of pairs (g_i_, g_j_) in *G* where g_i_ = 1 and g_j_ = 0  Append *AUC* to *A*  **End**  **Output:** *A,* List of length R containing AUROC values |


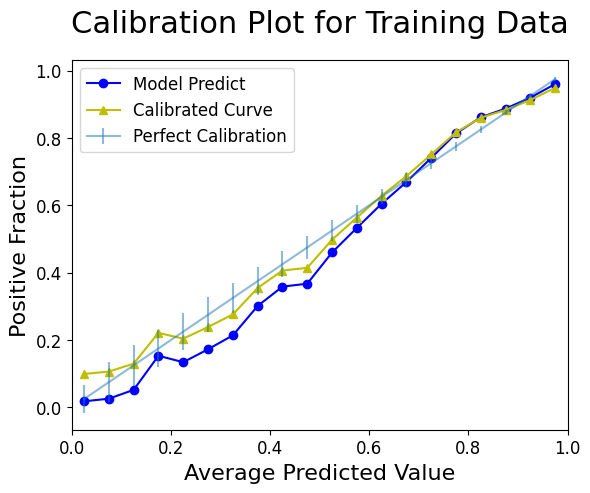


Figure S1: A calibration plot of the training data. The circle line is the uncalibrated model predictions, whereas the triangle line is the Platt scaled predictions. The green line shows the theoretical

perfect calibration, with the error bars showing the 95% confidence interval.
